# Structural Vulnerability Among Migrating Women and Children Fleeing Central America and Mexico: The Public Health Impact of “Humanitarian Parole”

**DOI:** 10.3389/fpubh.2015.00163

**Published:** 2015-06-24

**Authors:** Elizabeth Salerno Valdez, Luis A. Valdez, Samantha Sabo

**Affiliations:** ^1^Family and Child Health, Mel and Enid Zuckerman College of Public Health, The University of Arizona, Tucson, AZ, USA; ^2^Health Promotion Sciences, Mel and Enid Zuckerman College of Public Health, The University of Arizona, Tucson, AZ, USA

**Keywords:** structural vulnerability, humanitarian parole, immigration, detention, US–Mexico border

## Abstract

Since October 2013, US Customs and Border Patrol has apprehended 15,979 families on the Southwest Border of the US. Daily, migrating women and children from Mexico and Central America that qualify for *humanitarian parole* are released from immigration detention to a humanitarian aid organization in Southern Arizona. After several days in detention facilities, these families arrive tired, hungry, dehydrated, and with minimal direction regarding their final destination, and adherence to the parameters of their parole. *Project helping hands* (PHHs) utilizes a network of volunteers to provide the women and children with food, water, clothing, hygiene products, hospitality, and legal orientation. The aim of this assessment was to document the experiences of families granted humanitarian parole through the lens of structural vulnerability. Here, we apply qualitative methods to elicit PHH lead volunteer perspectives regarding the migration experience of migrating families. Using inductive analysis, we found six major themes emerged from the qualitative data: *reasons for leaving*, *experience on the journey*, *dehumanization in detention*, *family separation*, *vulnerability*, and *resiliency*. These findings elucidate the different physical and psychological distresses that migrating families from Mexico and Central America experience before, during and after their arrival at the US–Mexico border. We posit that these distresses are a result of, or exacerbated by, structural vulnerability. Structural vulnerability has life-long health implications for a sub-population of young mothers and their children. The number of migrating families who have experienced traumatic events before and during their migration experience continues to expand and thus warrants consideration of mental health surveillance and intervention efforts for these families. More public health research is needed to better understand and combat the health challenges of this growing population.

## Introduction

Since October 2013, US Customs and Border Patrol (USCBP) has apprehended 15,979 families on the Southwest Border of the US (1). Approximately 1,663 families traveling mostly from Mexico and Central have been apprehended in the Tucson sector of the border (1). After spending several days in detention, qualifying families are released to the local bus station to continue travel and reunite with their families in the US. However, due to language and cultural barriers, it is difficult for most families to navigate the national bus system. In the absence of federal or state involvement, humanitarian aid organizations have come to the aid of these families.

Multiple reasons exist for the current surge of Mexican and Central American families arriving at the US–Mexico border. Persistent economic deprivation and the resulting inability of families to adequately support themselves, as well as interminable violence are pushing people north. Recent studies indicate that the primary reason that people leave Guatemala, El Salvador, and Honduras is forced child recruitment into gangs, drug- and gang-related violence, gender-based violence, and extortion (2, 3). Violence is compounded by the lack of economic opportunities, lack of access to quality education, and the subsequent inability for families to support themselves financially in their home countries (4).

The government response to this surge is humanitarian parole, which is one of many temporary protection programs offered by the United States Citizenship and Immigration Services (USCIS). US temporary protection programs are dependent on parole, which is the primary avenue for temporarily admitting individuals into the US for extenuating circumstances, including catastrophic weather-related emergencies or violent conflict in sending countries. Humanitarian parole is a discretionary authority used sparingly in situations to grant entry to individuals who would otherwise be inadmissible into the US. The strict parameters of parole require the parolee to report to an immigration and customs appointment within 1–3 months at their final US destination, whereby their immigration status will be re-assessed. Parolees must leave the US before their parole expires, usually within 12 months, unless they gain a more permanent status given their specific circumstances (5).

In order to qualitatively assess the potential impact of the experiences of these families, we elected to assess this problem through the lens of structural vulnerability (6). The concept of structural vulnerability stems from Galtung’s (7) structural violence, which he defined as “the indirect violence built into repressive social order creating enormous differences between potential and actual human realization.” Kohler and Alock (8) explain that physical violence is when an actor (e.g., number of armed men) uses an instrument (e.g., ammunition) to cause a violent output (e.g., number of persons killed). They explain structural violence as systemic violence caused by structural attributes such as maldistributive policy that differentially allocates resources based on class oppression and economic injustice (6, 8). Alternatively, structural vulnerability was conceptualized to be more inclusive, encompassing maldistributive systems that are based on race, ethnicity, gender, and culture (6). Moreover, structural vulnerability refers to a positionality that imposes physical and emotional suffering on specific population groups and individuals in patterned ways. This positionality is a result of class-based economic exploitation and cultural, gender/sexual, and racialized discrimination (6). Furthermore, vulnerability results from an individual’s place in a hierarchical social order and its networks of power relationships and subsequent effects (9, 10). Currently, there is no systematic documentation of the demographics, immigration trajectory, and health status of these women and children. Therefore, the aim of this assessment is to document the experiences of families granted humanitarian parole through the lens of structural vulnerability.

## Materials and Methods

### Setting and participants

Investigators from the University of Arizona Mel and Enid Zuckerman College of Public Health partnered with p*roject helping hands (*PHHs) for this study. PHH provides services to migrating families who have been arrested and released by USCBP from short-term detention facilities in southern Arizona. Services include hospitality, food, water, clothing, basic medical attention, and instruction on the intercity bus system. In consultation with our research partner PHH and in order to protect the anonymity and privacy of the migrating women and children, researchers used purposive sampling to interview information-rich key informants, specifically lead PHH volunteers who serve migrating families (11). Lead volunteers hold a supervisory position, are the most likely to consistently interact with migrating families, and are required to speak Spanish fluently. PHHs volunteer coordinator provided the names and contact information of 20 lead volunteers. The investigators attempted to contact all volunteers via email on three separate occasions. Willing individuals replied to the investigators with their availability. A disclosure statement was provided to all volunteers. Face-to-face interviews took place in locations in the local community per the request of the participant. One interview was conducted over the phone. Interviews varied in length from 30 to 90 min. Data were gathered until empirical saturation was reached, i.e., participant responses ceased to vary greatly across questions (11).

Due to the non-generalizable nature of the data gathered, the authors submitted an exemption from human subject’s research to the University of Arizona’s Internal Review Board (IRB) and were approved on the grounds that non-generalizable data does not constitute as human research as defined by federal regulations.

### Interview guide

A semi-structured interview guide (Table 1) was designed to ascertain PHH volunteer perspectives of the migration experience, from departure from their home country to their arrival at PHHs. The open-ended interview questions were developed based on a literature review and consultation with border health and transnational migration field experts. The interview moderator guide was designed to elicit the following information: motivations for leaving the home country, the migration experience, experiences while in detention, PHH services and delivery, and migrating families’ health challenges.

**Table 1:** **Interview moderator guide**.

Domain	Questions
Reasons for leaving	What are some of the reasons that people are leaving their home country?
Experience on the journey	What are some of the stories that people have told you about the journey?
Experience in detention	Can you tell me about what the families may experience during detention, before arriving at PHH?
Family separation	Were there any health challenges of the women and children that you have served as a PHH volunteer?

### Data analysis

Interviews were audio recorded and transcribed. Two researchers with qualitative research experience independently read and coded the data for major themes using NVivo 7 Software (12). The researchers then compared notes on themes and through a process of consensus agreed on major and minor sub themes and developed a code book (Table 2). These same researchers again reviewed the transcripts and coded for the major and sub themes based on the codebook developed. Through face-to-face discussion, researchers developed a code memory to summarize each major and minor theme and identified illustrate quotes that best represented the themes encountered (13).

**Table 2:** **Qualitative codebook**.

Major themes	Sub themes	Definition	Sample quotes
Reasons for leaving	Escaping violence	Types of violence that motivate migrating families to leave home	*“He said ‘these bad men came and they took me and they put threads around my fingers and then they pulled them and put them against a tree, and then a big truck came and went through the threads and it cut my fingers off. I ran home and I was bleeding and bleeding and they told me to tell my mom that she was going to be killed and me too because we didn’t have anything more to give them. My mom was screaming and screaming and she just took me and we ran’”*
	Economic deprivation	Examples of economic deprivation influencing migration	*“I want to provide an opportunity for my children to get educated, to get a good job cause the only thing that they’re going to end up doing is getting into drugs and the gangs and they won’t do anything good in their lives if they stay”*
Experience on the journey	The train or the bus	Migrating families use different modes of transportation to reach the US depending on their social, material, and human capital	*“The people who get booted off the bus and the people who ride the top of the train are [going to] be the most vulnerable to begin with*…*”*
	Blending in	Success on the journey is partially dependent on how well migrating families blend in with Mexican nationals	*“But another way to do is to blend in more or less. So one woman, was actually a Honduran woman, she knew that like the government was [going to] get on the bus and be looking for suspicious looking Central Americans. So she gets on the bus and there’s the Central Americans are in the back of the bus and they are cowering and avoiding eye contact and such. So she decides to sit in the very front seat and whenever the police would get on would stand a very straight and would look at them straight in the eyes, just nod at them and they would walk right by her to the back to the back of a bus and grab the Central Americans and throw them off the bus”*
	Risk at the border	Migrating families encounter various types of risk at the border	*“[The coyotes] left with all of their money. They took all their money* … *and they are put up in a hotel and they’ll come and feed them whenever they can so they can go days without eating”*
	A confusing journey	Migrating families may lack resources, knowledge, language skills, and experience; thereby creating a sense of confusion about aspects/stages of the journey	*“For people who don’t, or aren’t familiar with this area, it’s desolate and they don’t know where they are”*
Dehumanization in detention		Participants discuss the dehumanizing experiences and/or conditions that migrating families endured while in detention facilities	*“Everyone says that the jail is way too cold and they sleep on floors they give them these like aluminum blankets and they feed them frozen burritos, which is like the worst thing you can have if you’re dehydrated from being on the desert*…*”*
Family separation		Migrating families separated during migration or at apprehension by immigration and customs	*“…her husband got detained and ended up somewhere so she was counting on him to help her with the kids and he was detained”*
	Vulnerability	Participants report the varying degrees of vulnerability before, during, and after the migration journey	*“I think the scariest thing is that they arrive almost exclusively from ICE from what we know with no dollars no money okay so they have to make the trip across the country with no basically to me that would be very scary to be a mom traveling with young kids and not have any money and be going from Arizona to Pennsylvania or Rhode Island”*
	Resiliency	Participant perceptions regarding the resiliency demonstrated by migrating families	*“…I see these and they’re not fragile these women are like incredibly resilient”*

## Results

Interviews were conducted with eight lead volunteers of PHHs. Six of the eight volunteers were female, and two were male. Ages in years ranged from the mid 20s to late 60s. Six of the eight volunteers were born in the US, and all but one of the volunteers was fluent in Spanish. All volunteers had been volunteering with PHH for a minimum of once per week for 4 months. Pseudonyms were assigned to each participant for de-identification purposes.

Six major themes emerged from the qualitative data: (1) reasons for leaving, (2) experience on the journey, (3) dehumanization in detention, (4) family separation, (5) vulnerability, and (6) resiliency.

### Reasons for leaving

#### Escaping Violence

In discussing why migrating families were fleeing their home countries, volunteers cited escaping violence as a primary cause for migrating north. Specifically, volunteers mentioned the pervasive drug violence affecting families and small businesses. Drug cartels demand money and resources, and when the families no longer have something to give, the cartels threaten them and their family members. Volunteers recalled mothers whose children had been recruited by drug cartels, and out of desperation, they gathered their belongings and fled. Volunteers stated that females often reported leaving due to domestic violence or sexual abuse by the men in their lives. Other reasons included extortion by government officials and police, kidnappings, and the high prevalence of crime in general. In one extreme case, a volunteer recalled the story of an 8-year-old boy from Honduras:

At that moment the little boy came and he had a little bag of cookies and he offered one to me and I noticed that four of his fingers were missing. I didn’t say anything. The little boy noticed that I was looking and he told me the story like matter of fact. He said, “these bad men came and they took me and they put threads around my fingers and then they pulled them and put them against a tree, and then a big truck came and went through the threads and it cut my fingers off. I ran home and I was bleeding and bleeding and they told me to tell my mom that she was going to be killed and me too because we didn’t have anything more to give them. My mom was screaming and screaming and she just took me and we ran.”

#### Economic Deprivation

Volunteers also cited economic deprivation as a reason, stating that many migrating families could no longer provide for their children with the current economic situation at home. Many families expressed that they needed better jobs with more pay in order to send their children to school.

One of the ladies said, “I want to provide an opportunity for my children to get educated, to get a good job cause the only thing that they’re going to end up doing is getting into drugs and the gangs and they won’t do anything good in their lives if they stay.”

### Experience on the journey

#### The Train or the Bus

The two overarching modes of transportation that migrating families used to arrive at the US–Mexico border from the southern border of Mexico were either by train or by bus. The trajectory through Mexico is heavily dependent on material, social, and human capital. Volunteers stated that those with sufficient means took the bus, which is generally safer than travel aboard the roof of a train. Their point of departure determined whether migrating families will either arrive at the Mexican border with Texas or Arizona. The large majority of those that arrive at the PHH house travel the route to the city of Agua Prieta, which borders Douglas, AZ, USA. The alternative mode of transportation is known as “La Bestia” (The Beast), a name that was earned by the slow moving cargo trains that cross Mexico from south to north. Migrating families told volunteers about having to remain awake on the top of the train, for risk of falling off while asleep. One volunteer reported meeting a young woman with a black eye and scratches all over her face that had been hit by a tree limb in the face while riding atop the train. As one of the volunteers mentioned:

Even if you aren’t subject to violence from the horrible people who are waiting to prey on people [riding on the train] there is still the reality of you riding on top of the cargo train for thousands of miles so that’s pretty awful.

The people who get booted off the bus and the people who ride the top of the train are [going to] be the most vulnerable to begin with you know the most destitute the most desperate and ones you maybe don’t speak the language as well are probably be the ones who get put into these more dangerous categories and are probably more will likely to be taken advantage of.

#### Blending In

Additionally, even when migrating families had the economic means to avoid the dangerous trajectory of the train, their success was heavily reliant on their abilities to speak Spanish and blend in with Mexican nationals. Immigration checkpoints in Mexico often required people to bribe federal officials, or be forced off the bus. One of the volunteers exclaimed:

So we only see the success stories here we see the people didn’t get kicked off the bus and the way they do that is either they’re a little wealthier, they have some pesos with them so that they can pay a bribe to the federales (federal police) … But another way to do is to blend in more or less. So one woman, was actually a Honduran woman, she knew that like the government was [going to] get on the bus and be looking for suspicious looking Central Americans. So she gets on the bus and there’s the Central Americans are in the back of the bus and they are cowering and avoiding eye contact and such. So she decides to sit in the very front seat and whenever the police would get on would stand a very straight and would look at them straight in the eyes, just nod at them and they would walk right by her to the back to the back of a bus and grab the Central Americans and throw them off the bus.

#### Risk at the Border

Travel on the bus eventually brought migrating families to the Sonora, Mexico border-area towns of Agua Prieta, Nogales, Altar, Sonoyta, or San Luis. While in these towns, migrating families were at risk of extortion and kidnapping, according to volunteers.

Another woman had one little kid and someone grabbed her before she got across the border someone grabbed her in Nogales and imprisoned her in some room that she had to eventually escape from out of the window, I suppose it was a coyote (human smuggler), she had like 1000 dollars or something and he took that money and imprisoned her and she didn’t know what his plans were for her but she finally got out a window.

Upon their arrival by bus or train, migrating families must pay a *coyote* to get across the border undetected. Volunteers described how migrating families were at the mercy of the *coyotes*, their law evasion strategies at the border, and their route through the arid Arizona desert.

[The coyotes] left with all of their money. They took all their money … and they are put up in a hotel and they’ll come and feed them whenever they can so they can go days without eating.

#### A Confusing Journey

Here, a volunteer described how confused migrating families become during the journey:

So they would be on the bus and they had there would be a series of payments made for those people and they would be riding buses until they get to the border area and in the border area depends on where they’re going to be crossed. If they spent the night in Altar Sonora they were in a rural area somewhat isolated, and they really had no idea was going on, they’re brought to the border, and for those who are familiar with Southern Arizona it’s a very open desert. For people who don’t, or aren’t familiar with this area, it’s desolate and they don’t know where they are.

### Dehumanization in detention

Several themes related to the *treatment, feeding, sleeping*, and *health status* of the migrating families during the apprehension and detention process. According to the volunteers, US Immigration and Customs Enforcement (ICE) attempted to process humanitarian parole as quickly as possible; however, setbacks in procedures forced families to spend anywhere from a couple of nights to several weeks in detention facilities. Volunteers also mentioned that families were often jailed in cold, windowless rooms, and were forced to sleep on the floor with only an emergency Mylar blanket for warmth. Volunteers described that the women and children often left ICE detention in a state of dehydration, not because of the journey prior to apprehension, but because they were not allowed to adequately hydrate while in detention. Moreover, they noted that most migrating families were not aware than the water available in the cells was potable, a fact that Border Patrol did not always share with their charges. Finally, the volunteers mentioned that the food provided to migrating families while in detention exacerbated their dehydration and made them sick.

Everyone says that the jail is way too cold and they sleep on floors they give them these like aluminum blankets and they feed them frozen burritos, which is like the worst thing you can have if you’re dehydrated from being on the desert … They feed them burritos, Austin crackers, and juices. They don’t eat the burritos, they eat the Austin crackers and the juices. So its funny when people were getting dropped off to us and they were dehydrated and they said they hadn’t eaten in a while I thought it was because they had been out in the desert but it was because they had been in the jail.

Many of them had no idea what was it just went through. The border patrol wakes people up at two in the morning and ‘processes them’. Meaning they interview them about the reasons why they left and they make some judgment legally about what that means at two in the morning. That’s normal procedure.

### Family separation

Another service provided by PHH was the location of missing family members. The parameters of humanitarian parole mandate that only one adult may remain to accompany any children, and any other adult is to be placed in short-term detention for deportation proceedings. This manifested itself in family separation; for example, a mother and father and their children may have been apprehended and the mother and children were released to PHH, while the father was detained. If they were not aware of where other members of the family were sent, PHH attempted to make contact with detention facilities to locate the men. Another situation that separated families is that some migrating families knew that as a male family member, if they crossed with women and children and were apprehended, they would be put in longer-term detention and separated from their families. As a result, that person may have attempted to enter the US separately. If this occurred, apprehended families were desperate to contact the missing members, and PHH worked to locate them with the Mexican or Guatemalan consulate. If they were able to communicate, this may have been an extreme relief, especially during the months of May through mid-September as that was the hot season and lives may be at stake in the desert.

These women take off. They just have to be so desperate to have to head off when she’s about to deliver a baby and then this other woman was there who had a 15 month old and she had a newborn and she was coming through and her husband got detained and ended up somewhere so she was counting on him to help her with the kids and he was detained.

### Vulnerability

Another emergent theme that arose from the interviews was the vulnerability of the migrating families both during their journey and upon their arrival in the US. Volunteers cited language barriers, lack of money, and a general lack of understanding of their immigration status as significant concerns for the migrating families. Language barriers were major obstacles for the migrating families as they moved through Mexico and into the US. Volunteers reported that the majority of families arriving at PHH were from rural areas of Guatemala and spoke indigenous languages. Their limited Spanish skills and inability to communicate added to their vulnerability and made it difficult to navigate through risky areas of Mexico. If they made it across the border, the inability to speak English added to their isolation when they were apprehended by the border patrol and detained by ICE. Understanding the process of apprehension, detention, the parameters of their humanitarian parole, and their future journey through the US became increasingly difficult when communication was impossible. Upon their arrival at PHH, the families were about to embark on yet another journey through an unfamiliar country, and may have spent up to 4 or 5 days navigating buses and stations before reaching their final destination. Another issue was that most migrating families arrived with no money for a variety of reasons. Some were robbed on the journey, while others fled imminent danger from cartel violence and had limited time for preparation and saving.

They’re just dropping these people off that don’t speak any English and don’t have any money and I thought that’s really horrible.

I think the scariest thing is that they arrive almost exclusively from ICE from what we know with no dollars no money okay so they have to make the trip across the country with no basically to me that would be very scary to be a mom traveling with young kids and not have any money and be going from Arizona to Pennsylvania or Rhode Island.

### Resiliency

While volunteers commented on the state of vulnerability of the migrating families, they also overwhelmingly remarked on the courage and resiliency of these individuals. They stated that in contrast to the manner in which immigrants are usually portrayed in the media as victims or as weak, their experience with migrating families has shown these individuals to be strong, resilient, and brave. They described them as individuals who are willing to do whatever it takes and overcome adversity in order to make a better life for them and their children.

There was a woman who walked, somewhere from Mexico, she had scratches all over her and she had a one year old little girl on her back. She was by herself and she wasn’t with a group. And they arrived just exhausted, just right on the edge. You wouldn’t know it because they’re just so tough, they wouldn’t have made it all the way here if they weren’t so tough but they were just right on the edge.

I would say that the people I’ve talked to do not fit the stereotype image that people coming of being super traumatized and you know almost shaky or anything like that. I see these and they’re not fragile these women are like incredibly resilient.

## Discussion

These findings elucidate the different physical and psychological distresses that migrating families from Mexico and Central America experience before, during and after their arrival at the Tucson sector of the US–Mexico border. We posit that these distresses are a result of, or exacerbated by, structural vulnerability. Structural vulnerability argues that inequality results from systemic political, economical, and material marginalization that contributes to oppression through gender, ethnic, and class-based discrimination (6, 9, 10). Migrating families find themselves in a particularly vulnerable situation due to the intersections of their status as economically marginalized indigenous women and children. Thereby, their status subjects them to class- and gender/sex-based exploitation, as well as culture- and race-based discrimination (6).

Our findings show that migrating families in question are fleeing situations characterized by structural vulnerability. Consistent with literature on the topic (2, 4, 6), we found that the primary motivation for women and children to flee their home country is persistent violence, specifically drug, gang, and gender-based violence, which largely occurs as a result of economic deprivation and maldistributive economic policy. Moreover, it has been found that children from Guatemala, El Salvador, and Honduras cited their primary reason for leaving their homes as forced child recruitment for gangs, and drug- and gang-related violence. Girls specifically have cited gender-based violence, including rape as a means of control by local gangs. Guatemalan children mentioned increasing poverty, poor agricultural yields, and unemployment as their reasons for leaving (2). Central American families overwhelmingly named deep structural conditions of economic security and chronic violence as reasons to migrate. Furthermore, the persistent threat of violence elicits fear and anxiety in children, increased risk of post-traumatic stress disorder (PTSD) and depressive symptoms in pre-adolescent and adolescent youth (14, 15).

In addition to experiencing violence at home, migrating families experience abuses while on the journey. Consistent with our findings, studies suggest that each year, hundreds of thousands of people from Guatemala, El Salvador, and Honduras attempt to cross Mexico, where they are regularly subjected to abuse, police brutality, extortion, rape, dismemberment, and death (16–18). However, violence does not cease upon their arrival at the US–Mexico border as migrants are vulnerable to violence from US immigration and law enforcement (19–21). Our findings illustrate the impact of USBP policy that separates, detains, and eventually deports men, and releases the women and children of the same family unit. While, there are many implications for all parties involved, research indicates that children suffer the greatest consequences from family separation. The immediate consequences of parental incarceration include traumatic separation, stigma, and isolation (22). Moreover, the effects of parental deportation are similar to that of other recognized negative childhood experiences, including family violence, a parent’s death, parental drug abuse, and neglect (19).

Turning to the issue of maltreatment while in custody of the USBP, volunteers reported that once they are apprehended, migrating families are subject to inhumane treatment, including verbal abuse, exposure to unbearably low temperatures, prohibited use of warm clothing, inadequate food, and limited water. Our findings are consistent with recent studies that found that migrants reported persistent abuse during USCBP apprehension, detention, and deportation proceedings (20, 21). Moreover, studies have shown that mistreatment during the apprehension and detention process may be psychologically distressful; data indicate that detention and adverse mental health are causally associated. Detention of asylum seeking migrants, even for short periods of time, results in acute psychological distress, the cause of new mental health disorders, and or the exacerbation of existing conditions (23–25). Additionally, children asylum seekers can suffer life-long psychological consequences as a result of detention, including acute psychological distress, anxiety depression, affective PTSD, and intentional self-harm (26–28).

Our findings elucidate that before, during, and after the migration journey, these migrating families are subject to class- and gender/sex-based exploitation, as well as culture- and race-based discrimination (6), thereby defining them as a structurally vulnerably population. It is important to note that their structural vulnerability is a result of systemic issues without simple solutions, and without broad-based political, economical, and social change, the US will not likely see a decrease in migration from these countries in the near future. For this reason, it is important to establish a public health framework for understanding these vulnerable populations as they transition into US society. The current immigration status of humanitarian parole provides little protection to vulnerable families. We believe that given the circumstances from which they flee, migrating families merit a more permanent state of protection than what is currently offered. We recommend that the US expand its capacity to admit migrating families who are at risk of violence or harm in their home countries, specifically by establishing a non-immigrant “protection” visa that would be made available to members of vulnerable groups (29).

Based on our findings and supporting literature, we recommend that Tucson sector USCBP detention conditions drastically improve in order to prevent the perpetuation of trauma, and subsequent negative health outcomes among migrating families. Processing of detainees should be expedited in order to reduce the time that families spend in detention. If USCBP custody is to become more humane, detention facilities must meet basic health needs. Families must have access to water in a sanitary manner and in sufficient quantities to ensure safe hydration, access to adequate foods that to not exacerbate dehydration, and adequate access to medical care. Moreover, detention facilities should have more adequate regulation of temperatures, allowance of more than one article of clothing per person in order to promote the recuperation and not exacerbate conditions of physical exhaustion and emotional trauma. Additionally, policies that promote the necessary separation of families should be eradicated, as they only serve to distress families and perpetuate trauma (17, 18, 22).

We recognize that this study has some limitations. Although we believe our methods provided adequate contextual understanding, second-hand inquiry limits the data given that we were unable to interview families directly. However, collecting volunteer perspectives was also a strength of the study, given that they are front-line humanitarian aid volunteers that work one on one with migrating families. Another potential limitation was the inclusion of data from a participant who was not fluent in Spanish. However, the researchers considered the contributions of this participant to be meaningful due to her consistent interactions with migrating families and simultaneous interpretation by Spanish-speaking volunteers. Finally, interviews were conducted with PHH volunteers serving only one site; thereby, the findings and related implications cannot be generalized to other migrating families that are crossing and being processed at other points of the US–Mexico border without further research.

Structural vulnerability has resulted in life-long health implications for a sub-population of young mothers and their children. The number of migrating families who have experienced traumatic events before, and during their migration experience continues to expand and thus warrants consideration of mental health surveillance and intervention efforts for these families. Although immigration policy-based solutions are beyond the scope of this article, we recognize that immigration and USCBP policy has critical public health implications, and therefore, should be considered in future investigations. Additional public health research is needed to better understand and combat the health challenges of this growing vulnerable population.

## Conflict of Interest Statement

The authors declare that the research was conducted in the absence of any commercial or financial relationships that could be construed as a potential conflict of interest.
